# Randomized Prospective Study Comparing Erythromycin, Amoxicillin, and Clindamycin for the Treatment of *Chlamydia trachomatis* in Pregnancy

**DOI:** 10.1155/S1064744995000020

**Published:** 1995

**Authors:** Mark A. Turrentine, Lisa Troyer, Bernard Gonik

**Affiliations:** 1 Department of Obstetrics, Gynecology, and Reproductive Sciences University of Texas Health Science Center—Houston 6431 Fannin Suite 3.204 Houston TX 77030 USA; 2 Department of Obstetrics and Gynecology University of Texas Medical Branch at Galveston Galveston TX USA; 3 Department of Obstetrics and Gynecology Wayne State University School of Medicine Detroit MI USA

## Abstract

*Objective:* The purpose of this study was to compare the efficacy and side effects of erythromycin, amoxicillin, and clindamycin in eradicating *Chlamydia trachomatis* from the lower genital tract of pregnant women.

*Methods:* A total of 174 women at <36 weeks gestation with positive cervical cultures for *C. trachomatis* were enrolled. Patients were assigned in a randomized prospective fashion to either erythromycin (500 mg q.i.d, for 7 days), amoxicillin (500 mg t.i.d, for 7 days), or clindamycin (600 mg t.i.d, for 10 days). Six women elected not to participate and 8 patients were lost to follow-up, leaving 53 patients in the erythromycin group, 55 patients in the amoxicillin group, and 52 patients in the clindamycin group. All sexual partners of the enrolled women were offered doxycycline (100 mg b.i.d. for 7 days) and patients were instructed to use barrier contraception until treatment was complete.

*Results:* All 3 medications were effective agents for the treatment of antenatal *C. trachomatis* infection with treatment efficacies of 96%, 94%, and 98% for the erythromycin, amoxicillin, and clindamycin groups, respectively. When the antibiotic groups were compared, no statistically significant differences were noted in intolerance. However, the differences in the incidence of gastrointestinal symptoms between erythromycin and amoxicillin and/or clindamycin were significant (P < 0.05).

*Conclusions:* These findings suggest that 1) all 3 antibiotic regimens are efficacious, 2) erythromycin has a higher incidence of side effects, and 3) amoxicillin or clindamycin are reasonable alternatives for the treatment of *C. trachomatis* in pregnant patients unable to tolerate erythromycin.


Infectious Diseases in Obstetrics and Gynecology 2:205–209 (1995)
© 1995 Wiley-Liss, Inc.

### Randomized Prospective Study Comparing Erythromycin, Amoxicillin, and Clindamycin for the Treatment of *Chlamydia trachomatis* in Pregnancy

**Mark A. Turrentine, Lisa Troyer, and Bernard Gonik**

*Department of Obstetrics, Gynecology, and Reproductive Sciences, University of Texas Health Science Center—Houston, Houston (M.A.T.), and Department of Obstetrics and Gynecology, University of Texas Medical Branch at Galveston, Galveston (L.T.), TX; and Department of Obstetrics and Gynecology, Wayne State University School of Medicine, Detroit, MI (B.G.)*

### Abstract

***Objective:*** **The purpose of this study was to compare the efficacy and side effects of erythromycin, amoxicillin, and clindamycin in eradicating *Chlamydia trachomatis* from the lower genital tract of pregnant women.**

***Methods:*** **A total of 174 women at <36 weeks gestation with positive cervical cultures for *C. trachomatis* were enrolled. Patients were assigned in a randomized prospective fashion to either erythromycin (500 mg q.i.d. for 7 days), amoxicillin (500 mg t.i.d. for 7 days), or clindamycin (600 mg t.i.d. for 10 days). Six women elected not to participate and 8 patients were lost to follow-up, leaving 53 patients in the erythromycin group, 55 patients in the amoxicillin group, and 52 patients in the clindamycin group. All sexual partners of the enrolled women were offered doxycycline (100 mg b.i.d. for 7 days) and patients were instructed to use barrier contraception until treatment was complete.**

***Results:*** **All 3 medications were effective agents for the treatment of antenatal *C. trachomatis* infection with treatment efficacies of 96%, 94%, and 98% for the erythromycin, amoxicillin, and clindamycin groups, respectively. When the antibiotic groups were compared, no statistically significant differences were noted in intolerance. However, the differences in the incidence of gastrointestinal symptoms between erythromycin and amoxicillin and/or clindamycin were significant ($P < 0.05$).**

***Conclusions:*** **These findings suggest that 1) all 3 antibiotic regimens are efficacious, 2) erythromycin has a higher incidence of side effects, and 3) amoxicillin or clindamycin are reasonable alternatives for the treatment of *C. trachomatis* in pregnant patients unable to tolerate erythromycin.**
© 1995 Wiley-Liss, Inc.

Key words
Chlamydial infections, antenatal antibiotic therapy, pregnancy

*Chlamydia trachomatis* is the most frequently encountered sexually transmitted disease in the United States[1] and is subsequently seen in pregnancy with a high prevalence. It has been estimated that each year more than 155,000 infants are born to women infected with *C. trachomatis*.[1] The vertical transmission of *C. trachomatis* can result in conjunctivitis or pneumonia in up to 50% or 20% of exposed infants, respectively.[2] Although erythromycin has traditionally been the recommended antibiotic to treat *C. trachomatis* during pregnancy,[1] it is poorly tolerated due to gastrointestinal side

**Address correspondence/reprint requests to Dr. Mark A. Turrentine, Department of Obstetrics, Gynecology, and Reproductive Sciences, University of Texas Health Science Center—Houston, 6431 Fannin, Suite 3.204, Houston, TX 77030.**
**Presented at the 42nd Annual Clinical Meeting of the American College of Obstetricians and Gynecologists, Orlando, FL.**

Received July 29, 1994

Accepted November 2, 1994

Clinical Study effects. Investigations into alternative antibiotic therapies have shown that amoxicillin[3–5] and clindamycin[6] are efficacious in treating antenatal *C. trachomatis* infections. Currently, the Centers for Disease Control (CDC) guidelines recommend either erythromycin or amoxicillin for treatment of chlamydial infection in pregnancy.[1] No comparison of erythromycin, amoxicillin, and clindamycin in the treatment of *C. trachomatis* infection during pregnancy has been reported. We conducted the current study to determine, using a randomized prospective design, the comparative efficacy of erythromycin, amoxicillin, and clindamycin in eradicating *C. trachomatis* from the lower genital tract of pregnant women.

### SUBJECTS AND METHODS

The study was approved by the institutional review board of the University of Texas Health Science Center—Houston. Women with cervical cultures positive for *C. trachomatis* before 36 weeks gestation were eligible with the following exclusion criteria: sensitivity to any of the study medications, persistent gastrointestinal symptoms, or antibiotic therapy after screening and before enrollment. A randomized prospective trial was conducted, enrolling patients from the University of Texas Hospital obstetrical clinic between July 1991 and September 1993.

After informed consent was obtained, each eligible patient was randomly assigned to 1 of 3 treatment groups: erythromycin-base tablets, 500 mg q.i.d. orally for 7 days; amoxicillin capsules, 500 mg t.i.d. orally for 7 days; or clindamycin tablets, 600 mg t.i.d. orally for 10 days. The randomization was accomplished by computer-generated assignment, and the unlabeled medications were dispensed by the hospital pharmacy to prevent the health-care team from learning the assigned medication. The partners of all participants received doxycycline, 100 mg b.i.d. orally for 7 days. The subjects were asked to abstain from sexual intercourse or to use barrier contraception until both members of the couple had completed their courses of medication. The primary outcome variable was defined as having completed the course of medication with a negative test-of-cure.

Four weeks after completion of the study medication, the patients had test-of-cure cultures from the cervix. All patients were asked to return to the clinic with their medication bottles for a pill count. In addition, each patient was asked to complete a questionnaire to document if 1) all medication was taken, 2) any symptoms were noted while taking the medication, and 3) her partner was treated.

The patients had test-of-cure cultures from the cervix. All specimens were obtained from the endocervical canal with plastic-handled, cotton-tipped swabs after the ectocervix had been cleared of secretions. Specimens for *C. trachomatis* were placed immediately into transport media and processed within 12 h. They were inoculated into cycloheximide-treated McCoy cells in shell-vial monolayers, incubated for 48 h, fixed with ethanol, and stained with fluorescein-conjugated monoclonal antibody to *C. trachomatis* (Microtrak, Syva Co., Palo Alto, CA). The inclusions were detected by fluorescence microscopy. Any patient who had a positive test-of-cure was contacted by telephone to assess if the medication had been taken appropriately, if her partner had been treated adequately, and if unprotected intercourse had occurred after enrollment or before the test-of-cure.

Statistical comparisons were performed using $\chi^2$, Fisher exact test, or t-test when appropriate; $P < 0.05$ was regarded as significant.

### RESULTS

A total of 174 eligible patients were enrolled in the study (prevalence 6.4%); 6 women elected not to participate. Of the 168 patients enrolled, 56 women received erythromycin, 57 received amoxicillin, and 55 received clindamycin. Eight patients were lost to follow-up, 3 in the erythromycin, 2 in the amoxicillin, and 3 in the clindamycin group, and were excluded from the analysis. The demographic characteristics of the patients in each treatment group are shown in Table 1. There were no statistically significant differences in age, racial distribution, gravidity, gestational age, or number of days to test-of-cure among the groups.

The success of the regimen was defined as completing the course of medication and having a negative test-of-cure culture. Forty-five of the 53 (85%) evaluable women assigned to the erythromycin group successfully completed their regimens, compared with 50 of the 55 (91%) women in the amoxicillin group and 47 of the 52 (90%) women in the clindamycin group (Fig. 1). These differences were not statistically significant.

**TABLE 1. Demographic characteristics at enrollment[a]**

| | Erythromycin | Amoxicillin | Clindamycin |
|---|---|---|---|
| Age (years) | 20.3 ± 3.8 | 20.2 ± 3.4 | 18.6 ± 2.8 |
| Race (% nonwhite) | 64.4 | 68.0 | 74.5 |
| Gravidity | 2.2 ± 1.2 | 2.4 ± 1.2 | 1.9 ± 1.0 |
| EGA (weeks) | 24.3 ± 6.6 | 24.9 ± 6.3 | 22.9 ± 6.2 |
| Number of days to test-of-cure | 35.9 ± 8.7 | 31.1 ± 9.3 | 34.7 ± 9.0 |

[a]Results are mean ± SD. No differences were statistically significant. EGA = estimated gestational age.

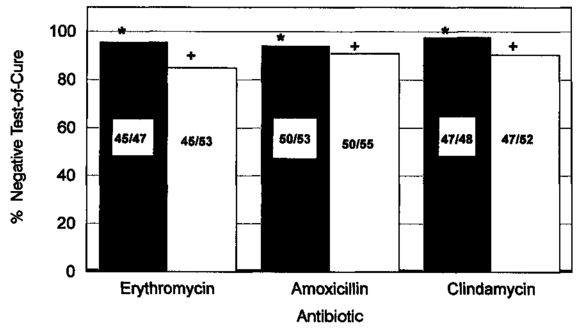

No differences were statistically significant.
* Negative test-of-cure for patients completing a course of medication.
+ Number of patients completing the course of medication and having a negative test-of-cure out of those assigned to the regimen.

Fig. 1. Treatment efficacy.

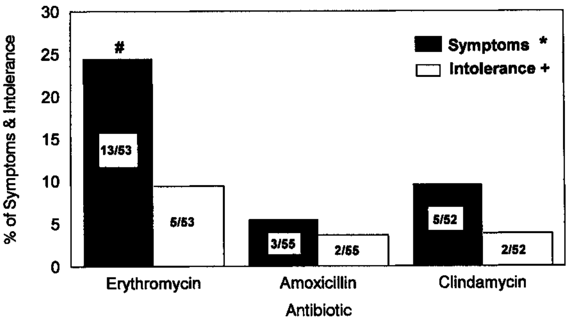

### P < .05 for symptoms

* Symptoms include patients who were intolerant to the medication.

+ Side effects severe enough to result in discontinuation of therapy.

Fig. 2. Symptoms and intolerance.

Of the 53 patients in the erythromycin group, 1 developed an allergic reaction to erythromycin although she had no history of an allergy. In addition, 13 women had gastrointestinal side effects due to the erythromycin which resulted in 5 participants discontinuing the therapy. Of the remaining 47 patients, 45 (96%) had negative cervical cultures after completing therapy. The 2 women who failed treatment in the erythromycin group reported having completed their medication. One of the failures admitted that her partner had not been treated with doxycycline.

In the amoxicillin group, 55 patients were evaluated. Three women had gastrointestinal side effects due to amoxicillin which resulted in 2 discontinuing the therapy. Fifty of the 53 (94%) patients completing therapy had subsequent negative cervical cultures. All 3 women who failed treatment in the amoxicillin group reported having completed their treatment. Although all 3 women also reported that their partners were not treated, 2 denied having intercourse with their respective partners since beginning their treatment.

In the clindamycin group, 52 patients were evaluated. Of these, 2 women developed an allergic reaction to clindamycin although neither had any history of an allergy. Five women had gastrointestinal side effects due to clindamycin which resulted in 2 discontinuing the therapy. Forty-seven of 48 (98%) patients completing therapy had subsequent negative cervical cultures. The woman who failed treatment in the clindamycin group reported completion of her treatment and compliance of her partner with the doxycycline.

All 3 medication regimens, erythromycin, amoxicillin, and clindamycin, were effective for the treatment of antenatal *C. trachomatis* infection. Figure 1 shows that, with cure defined as a negative test-of-cure culture for a patient completing treatment, the treatment efficacies were 96%, 94%, and 98% for the erythromycin, amoxicillin, and clindamycin groups, respectively. These differences were not statistically significant.

No life-threatening side effects were encountered in any of the antibiotic groups. Three patients developed a rash to the medication (erythromycin = 1 and clindamycin = 2) which required the discontinuation of therapy. Figure 2 shows the incidence of intolerance (defined as side effects, such as abdominal pain, emesis, and diarrhea, severe enough to result in the discontinuation of therapy) and symptoms for the 3 antibiotic regimens. No statistically significant differences were noted in intolerance among all antibiotics. No differences in the incidence of gastrointestinal symptoms due to the treatment medication between the amoxicillin or clindamycin group were significant. However, the differences in the incidence of gastrointestinal symptoms between erythromycin and amoxicillin and/or clindamycin were significant ($P < 0.05$).

### DISCUSSION

Traditionally, the recommended drug for the treatment of antenatal *C. trachomatis* infections has been the macrolide erythromycin.[1] However, this medication is typically poorly tolerated due to gastrointestinal side effects. Amoxicillin[3–5] and clindamycin[6] have been recommended as alternative regimens for the treatment of *C. trachomatis* in pregnancy. Several studies[3–5] have shown that amoxicillin, 500 mg t.i.d. for 7 days, is as effective as erythromycin in eradicating antenatal *C. trachomatis*. Treatment efficacies of 82–98% have been shown.[3–5] Our result of 94% agrees with these previous findings.

Clinical trials using clindamycin for the treatment of *C. trachomatis* have been limited. Bowie et al.[7] reported that a 7-day clindamycin regimen of 600 mg t.i.d. was only partially effective for the treatment of chlamydial urethritis in men. However, Campbell and Dodson[8] showed an 85% cure rate in nonpregnant women with *C. trachomatis* who took clindamycin, 450 mg q.i.d. for 10 days. A similar treatment efficacy (94%) was demonstrated by Alger and Lovchik[6] with antenatal *C. trachomatis* infections in women who took clindamycin, 450 mg q.i.d. for 14 days. In the current study, a 10-day regimen of clindamycin was utilized, and 98% of the women who completed the therapy had negative tests-of-cure.

No previous study has compared both amoxicillin and clindamycin with erythromycin for the treatment of antenatal *C. trachomatis*. Using a randomized prospective study, we demonstrated that either amoxicillin, 500 mg t.i.d. for 7 days, or clindamycin, 600 mg q.i.d. for 10 days, was an effective and well-tolerated regimen for the treatment of antenatal chlamydial infections. Approximately 94% of the patients taking amoxicillin and 98% of the patients taking clindamycin were cured. These rates are similar to previous reports of amoxicillin[3–5] and clindamycin[6] used for the treatment of antenatal chlamydial infections. A larger study would be necessary to exclude the possibility of β-error and the superiority of amoxicillin or clindamycin.

Using the CDC-recommended dosage of erythromycin for the treatment of antenatal *C. trachomatis*[1] resulted in a large number of patients (24.5%) experiencing gastrointestinal symptoms, which was statistically different from the amoxicillin (5.5%) and clindamycin (9.6%) groups. When these 2 treatments were compared with erythromycin, there was no significant difference in efficacy. Similar results have been reported in studies comparing erythromycin with amoxicillin[3–5] or clindamycin.[6] Although twice as many patients in the current study experienced gastrointestinal side effects from erythromycin (9.4%) which resulted in the discontinuation of therapy, this was not significantly different from the amoxicillin (3.6%) or clindamycin (3.8%) group. This finding is in contrast to that of Magat et al.,[4] who showed a statistical difference between erythromycin and amoxicillin with regard to intolerance.

Our findings suggest that all 3 antibiotic regimens are efficacious in the treatment of antenatal *C. trachomatis* infections. Therefore, because erythromycin has a higher incidence of side effects that may result in the discontinuation of therapy, either amoxicillin or clindamycin is a reasonable alternative for the treatment of *C. trachomatis* in pregnant patients unable to tolerate erythromycin.

### ACKNOWLEDGMENTS

The authors thank the pharmaceutical companies Parke-Davis, Lederle, and Upjohn for providing the treatment medications used in this study.

### REFERENCES

1. Recommendations for the prevention and management of *Chlamydia trachomatis* infections, 1993. MMWR 42:1–39, 1993.
2. Harrison HR, Alexander ER: Chlamydia infections in infants in infants and children. In Holmes KK, Mårdh PA, Sparling PF, et al. (eds): Sexually Transmitted Diseases. New York: McGraw-Hill, pp 811–820, 1990.
3. Crombleholme WR, Schachter J, Grossman M, Landers DV, Sweet RL: Amoxicillin therapy for *Chlamydia trachomatis* in pregnancy. Obstet Gynecol 75:752–756, 1990.
4. Magat AH, Alger LS, Nagey DA, Hatch V, Lovchik JC: Double-blind randomized study comparing amoxicillin and erythromycin for the treatment of *Chlamydia trachomatis* in pregnancy. Obstet Gynecol 81:745–749, 1993.
5. Silverman NS, Sullivan M, Hochman M, Womack M, Jungkind DL: A randomized, prospective trial comparing amoxicillin and erythromycin for the treatment of *Chlamydia trachomatis* in pregnancy. Am J Obstet Gynecol 170: 829–832, 1994.
6. Alger LS, Lovchik JC: Comparative efficacy of clindamycin versus erythromycin in eradication of antenatal *Chlamydia trachomatis*. Am J Obstet Gynecol 165:375–381, 1991.
7. Bowie WR, Yu JS, Jones HD: Partial efficacy of clindamycin against *Chlamydia trachomatis* in men with nongonococcal urethritis. Sex Transm Dis 13:76–80, 1986.
8. Campbell WF, Dodson MG: Clindamycin therapy for *Chlamydia trachomatis* in women. Am J Obstet Gynecol 162:343–347, 1990.
